# Functional interaction between plasma phospholipid fatty acids and insulin resistance in leucocyte telomere length maintenance

**DOI:** 10.1186/s12944-020-1194-1

**Published:** 2020-01-17

**Authors:** Yi Zhao, Binxia Wang, Guoqi Wang, Lixia Huang, Ting Yin, Xiaoxia Li, Xiuying Liu, Qingan Wang, Jinyun Jing, Jianjun Yang, Yuhong Zhang

**Affiliations:** 1 Department of Epidemiology and Health Statistics, School of Public Health and Management, Ningxia Medical University, 1160 Shengli Street, Xingqing District, Yinchuan, Ningxia Hui Autonomous Region People’s Republic of China; 2 Gansu Center for Disease Control and Prevention, No.371 Chengguan District, Lanzhou, Gansu China

**Keywords:** Plasma phospholipid fatty acids, HOMA-IR, Leukocyte telomere length, Interaction

## Abstract

**Background:**

Previous evidence suggests that plasma phospholipid fatty acids (PPFAs) and HOMA insulin resistance (HOMA-IR) are independently related to leukocyte telomere length (LTL). However, there is limited evidence of regarding the effect of their interaction on relative LTL (RLTL). Therefore, here, we aimed to determine the effect of the interaction between PPFAs and HOMA-IR on RLTL.

**Methods:**

We conducted a cross-sectional study, involving a total of 1246 subjects aged 25–74 years. PPFAs and RLTL were measured, and HOMA-IR was calculated. The effect of the interaction between PPFAs and HOMA-IR on RLTL was assessed by univariate analysis, adjusting for potential confounders.

**Results:**

In age-adjusted analyses, multivariate linear regression revealed a significant association of the levels of elaidic acid, HOMA-IR, monounsaturated fatty acids (MUFA) and omega-6 (n-6) polyunsaturated fatty acid (PUFA) with RLTL. After adjustment of age and gender, race, smoking, drinking, tea, and exercise, elaidic acid, and omega-3 (n-3) PUFA were negatively associated with RLTL, and HOMA-IR and n-6 PUFA were positively associated with RLTL. These associations were not significantly altered upon further adjustment for anthropometric and biochemical indicators. Meanwhile, the effect of the interaction of elaidic acid and HOMA-IR on RLTL was significant, and remained unchanged even after adjusting for the aforementioned potential confounders. Interestingly, individuals who had the lowest HOMA-IR and the highest elaidic acid levels presented the shortest RLTL.

**Conclusions:**

Our findings indicated that shorter RLTL was associated with lower HOMA-IR and higher elaidic acid level. These findings might open a new avenue for exploring the potential role of the interaction between elaidic acid and HOMA-IR in maintaining RLTL.

## Introduction

Leukocyte telomere length (LTL) is a simple and reliable biomarker of biological age [1], and it is influenced by dietary factors [2]. Recently, a large number of studies have explored the association between dietary factors and LTL, but their results are inconsistent [3]. For example, Tiainen et al. finds that total fat and saturated fatty acid intake are inversely associated with LTL in elderly men from Helsinki Birth Cohort Study [4], but that there is no association in Spanish children and adolescents [5]. A systematic review shows that, since much researches are based on population dietary surveys, investigation bias may be one of the dominating reasons for the inconsistency of results [2]. A previous study confirms that plasma phospholipid fatty acids (PPFAs) can be used as valid markers reflecting long-term intake of dietary fatty acids [6]. However, to our knowledge, only few studies have explored the relationship between PPFAs and LTL, and the result reveals that trans fatty acid (TFA) levels and, particularly palmitelaidic and linolelaidic acid, are likely negatively associated with telomere length [7].

Some case-control studies, on individuals clinically diagnosed with insulin resistance show, that insulin resistance is associated with chromosomal LTL, and a rise in insulin resistance is the primary reason for the acceleration of telomere shortening [8, 9]. A research also manifests that insulin resistance acts as an indicator of healthy aging in humans [10]. The HOMA-insulin resistance (HOMA-IR) index can be used to evaluate the insulin resistance of individuals [11]. However, the results of previous studies on HOMA-IR index and LTL are controversial due to difference in populations studied and analysis methods used [12, 13], and Sampson et al. finds telomere length is unrelated to insulin resistance in type 2 diabetes [14]. These conflicting findings suggest that the link between HOMA-IR index and LTL may not fit neatly into a simple paradigm.

Therefore, the aim of our study was to investigate whether PPFAs and HOMA-IR affect the relative LTL (RLTL) aged 25–74 years, and to further determine whether the interaction between PPFAs and HOMA-IR plays a role in RLTL modification.

## Methods

### Study design and selection of subjects

This study was conducted as a cross-sectional survey from 2008 to 2012 in the Qingtongxia County and Pingluo County, of Ningxia Hui Autonomous Region, China. Stratified cluster sampling was carried out to select two villages in each county. A total of 3064 subjects aged 25–74 years were recruited. Each participant underwent a structured in-person questionnaire interview about general demographic characteristics, behavioral lifestyles, and current disease status. Pregnant or breastfeeding women and patients of disease, such as coronary heart disease, diabetes mellitus, severe mental illness, infectious diseases, autoimmune diseases, and tumors were excluded. Height, weight, waist and hip circumference were measured by trained and qualified investigators. Sitting blood pressure was measured by electronic sphygmomanometer (Omron-HEM 7301-IT, China) and body mass index (BMI), waist-to-hip ratio (WHR) were calculated. We employed a mechanical sampling and voluntary principle to select blood samples, and a total of 1458 specimens were collected. We excluded people with aforementioned diseases based on the questionnaire, as well as those of people with missing data on questionnaire, anthropometric measuring and experiment, and finally, 1246 participants were included in the analysis. This study was approved by Ningxia Medical University ethics committee, and all participants were obtained written and verbal information about our study and gave written informed consent.

### Blood collection and laboratory tests

Participants were advised to fast for at least 8 h before blood sample collection. In the morning, specialized physicians drew 5 ml peripheral vein blood from the participants into a non-anticoagulant tube and 2 ml into an EDTA-anticoagulant tube. The blood samples in the EDTA-anticoagulant tubes were centrifuged and kept at − 80 °C for testing. The blood samples in the non-anticoagulant tubes were used for a series of laboratory measurements. Fasting plasma glucose was instantly detected by One Touch Ultra 2 (LifeScan, USA). Fasting plasma insulin was determined by enhanced chemiluminescence immunoassay (Tegke Xin Biotech Co., Ltd., Beijing, China). HOMA-IR was calculated using the following formula: [fasting plasma insulin (mU/L) × fasting plasma glucose (mmol/L)] / 22.5. Cholesterol and triglycerides were measured by enzymatic assay (CHOD-PAP, Roche Diagnostics GmbH). PPFAs were determined by gas chromatography (Agilent Technologies 6890 N, America).

### DNA extraction and RLTL

Genomic DNA on leukocytes was extracted from peripheral blood samples using the D3392–04 DNA blood mid kit (Bao Bioengineering Co., Ltd., Japan). The DNA concentration and purity were detected using the Biospec-nano instrument (Shimadzu, Japan), and OD260/OD280 was qualified between 1.6 and 1.9. RLTL was measured using a real-time fluorescence quantitative PCR (Bio-Rad, Germany) method previously described by Cawthon [15]. PCRs were carried out in separate 96-well plates, which were segmented into two parts: one for the telomeres (T) and one for the housekeeping gene 36B4 (S), and each plate must contained a reaction for the reference gene and a negative control. Cycling conditions for telomere amplification were as follows: 95 °C for 10 min to active the FastStart Enzyme (Bao Bioengineering Co., Ltd., Japan), denaturing 95 °C 15 s, annealing at 54 °C 2 min, with a total of 22 cycles. Cycling conditions for the 36B4 gene were as follows: the starting condition was the same as that for telomeres, but the annealing condition was 58 °C 2 min, with a total of 30 cycles. Finally, the relative T/S ratio, reflecting RLTL, was calculated through the ΔΔCt method, using the following equations: T/S = [2^Ct(telomere)^ / 2^Ct(36B4)^]^− 1^ = 2^-ΔCt^, RLTL = 2^-ΔCt^ (need checking sampling) / 2^-ΔCt^ (reference gene).

### Statistical analysis

Analyses were performed using the SPSS 23.0 statistical package, and a two-tailed *P-value* less than 0.05 indicated statistical significance. The normality of the distribution of the values of all variables was tested. Continuous variables were expressed as mean ± standard deviation (*SD*) in for normally distributed data and, as median with interquartile range (*IQR*, 25th ∼ 75th percentile) for non-normally distributed. Categorical variables were described as frequencies (percentages). Comparisons between the variables in different RLTL groups were performed using *ANOVA* or *Kruskal-Wallis H* tests for non-normally distributed data. Multivariate linear regression analyses for PPFAs, HOMA-IR index and RLTL were performed to exclude the influences of potential confounding variables. Then, elaidic acid and HOMA-IR were separately stratified into quintiles, and the means and 95% confidence intervals (*95%CI*) of RLTL were compared using *LSD*. In addition, to assess the effect the of interaction of elaidic acid and HOMA-IR on RLTL, univariate analysis was employed. Linear trend tests were used to show trend changes of elaidic acid and HOMA-IR index on RLTL.

## Results

The mean age of the 1246 subjects included in this study was 50.0 years (*SD* 11.8), and 59.4% of these subjects were female. In a preliminary analysis, the subjects were divided into tertiles according to RLTL. The tertile cut-offs were as follows: 0.645, 1.884, and general characteristics of the study participants were presented in Additional file 1: Table S1. As expected, there were significant differences in RLTL based on age in which the lowest tertile had a higher age compared with the middle and upper tertiles, suggesting that RLTL declined with increasing age (*P* < 0.001). Interestingly, WHR also showed similar trend. In addition, RLTL appeared to differ by tea consumption (*P* = 0.0036) and systolic blood pressure (SBP) (*P* = 0.0045), but not by gender, race, smoking, drinking, exercise, BMI, diastolic blood pressure (DBP) (*P* > 0.05).

### Metabolism indicators and PPFAs

In the lowest tertile group of RLTL, fasting plasma insulin, fasting plasma glucose and HOMA-IR were significantly lower (*P* < 0.001); in contrast, high-density lipoprotein cholesterol (HDL-C) was significantly higher (*P* < 0.001). However, the values of palmitic acid, stearic acid, elaidic acid, α-linolenic acid, arachidonic acid, saturated fatty acids (SFA), monounsaturated fatty acids (MUFA), and omega-3 (n-3) polyunsaturated fatty acids (PUFA) were higher in the lowest tertile group, and all of them decreased with an increase in RLTL (Additional file 1: Table S1).

### Associations between PPFAs, HOMA-IR and RLTL

Next, we applied linear correlation to explore the association of RLTL with PPFAs and metabolic indices. We found that RLTL was inversely associated with palmitic acid, stearic acid, elaidic acid, α-linolenic acid, arachidonic acid, SFA, MUFA, n-3 PUFA, and HDL-C; and it was positively associated with n-6/n-3, fasting plasma glucose, HOMA-IR; then other indicators were not associated with RLTL seemingly. These associations were not appreciably altered by adjustment for age (Additional file 1: Table S2).

To evaluate multivariate correlation, the relevant variables mentioned above were converted by log to meet the analytical conditions. The age-adjusted analyse revealed a vital association of the levels of elaidic acid, HOMA-IR index, MUFA and n-6 PUFA with RLTL. After adjustment for gender, race, smoking, drinking, tea, and exercise, elaidic acid and n-3 PUFA were found to be negatively associated with RLTL, and HOMA-IR index and n-6 PUFA were positively associated with RLTL. These associations were not appreciably altered by adjustment for other factors that might influence RLTL, including BMI, WHR, SBP, DBP, total cholesterol (TC), triglycerides (TG) and HDL-C (Additional file 1: Table S3).

### Effect of the interaction between elaidic acid and HOMA-IR index on RLTL

**Fig. 1:**
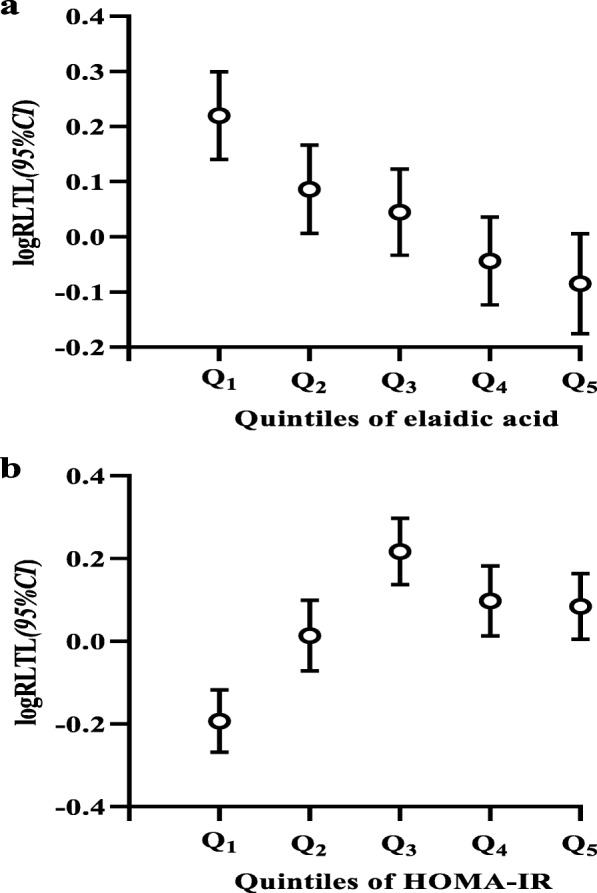
Difference in RLTL between quintile groups of elaidic acid (**a**) and HOMA-IR (**b**). Describes the differences in RLTL between quintiles groups of elaidic acid and HOMA-IR index. Quintile cut-offs were 50.22, 76.94, 95.30, 131.43 ng/mL for elaidic acid (**a**) and 0.77, 1.20, 1.44, 1.95 for HOMA-IR (**b**). The sbscissa represents the quintiles of elaidic acid (**a**) and HOMA-IR (**b**), and ordinate represents logRLTL (*95%CI*)

We tested for differences in RLTL between quintiles groups of elaidic acid and the HOMA-IR index. Notably, the higher elaidic acid content, the shorter was the RLTL (*P-*_*ANOVA*_ < 0.001, *P-trend* < 0.001). In subjects with more than 131.43 ng/mL of elaidic acid, the RLTL was 0.30 lower compared with those who were in the first quintile (Fig. 1 a). On the contrary, among subjects in the lowest HOMA-IR quintile group, the RLTL was 0.41 lower compared with those in the middle HOMA-IR quintile group (*P-*_*ANOVA*_ < 0.001, *P-trend* < 0.001) (Fig. 1 b). In addition, there were significant differences between the RLTL values in the lowest quintiles group compared with those in the other quintiles groups of elaidic acid and HOMA-IR (all *P* < 0.05).

When we evaluated potential effects of the interaction between elaidic acid and HOMA-IR index on RLTL, the subjects were divided into tertiles according to elaidic acid and HOMA-IR index. In the rude mode, the effects of elaidic acid, HOMA-IR index, and their combination were related to RLTL (*P* for interaction = 0.020). Particularly, the interaction associations were not greatly altered by adjustment for potential confounders (*P* < 0.05 for each interaction) (Additional file 1: Table S4).

**Fig. 2:**
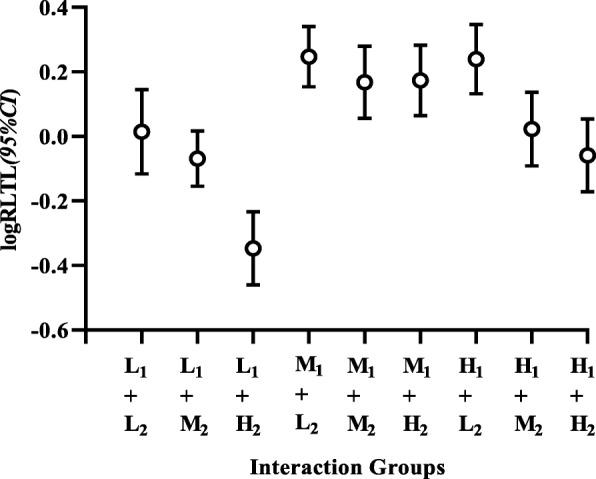
The effect of interaction between elaidic acid and HOMA-IR index on RLTL. Presents the effect of interaction between elaidic acid and HOMA-IR index on RLTL. RLTL values were classified according to categories of elaidic acid and HOMA-IR index. Cut-offs for elaidic acid were < 69.23 (L), 69.23 to 103.04 (M), and > 103.04 (H) ng/mL, and cut-offs for HOMA-IR were > 1.52 (H), 1.12 to 1.52 (M) and < 1.12 (L) (1: HOMA-IR index, 2: elaidic acid). The abscissa represents the interacton groups, and the ordinate represents logRLTL (*95%CI*)

Moreover, when dividing subjects according to the combination of elaidic acid and HOMA-IR index into three categories, those who simultaneously showed the highest elaidic acid and the lowest HOMA-IR index significantly presented the lowest RLTL compared to those of other groups. Then, considering the group with highest elaidic acid and the lowest HOMA-IR index as the reference, statistically differences were observed between the reference group and others (all *P* < 0.01). In addition, RLTL showed a decreasing trend with an increase in the elaidic acid level at different HOMA-IR index levels (Fig. 2).

## Discussion

In this cross-sectional study involving 1246 subjects from a rural Chinese population, we observed that the elaidic acid was negatively correlated with RLTL, and HOMA-IR index was positively correlated with RLTL. Particularly, the lowest HOMA-IR index and the highest elaidic acid level were associated with the shortest RLTL in our study.

### PPFAs and RLTL

TFAs are closely related to the occurrence of cardiovascular disease [16], and the underlying mechanism mainly involves that TFAs mediates damage and apoptosis of vascular endothelial cells by activation of death receptor pathways and mitochondrial pathways [17]. A previous study demonstrates that TFAs intake is positively associated with markers of systemic inflammation in women [18]; moreover, epidemiological and clinical studies reveal that a TFA-rich diet obviously increases the serum concentrations of high sensitivity C-reactive protein, interleukin-6, and tumor necrosis factor [18–20]. However, LTL ostensibly reflects the cumulated burden of inflammation and oxidative stress over an individual’s lifespan [21]. Chan et al. [22] finds that the intake of fats and oils is negatively associated with LTL in elderly females, and Mazidi et al. points out that TFA levels are likely negatively related to telomere length [7]. The findings of the present study were consistent with these findings, and indicated that TFAs are a risk factor for RLTL.

At present, the relationship between PUFA and LTL is controversial. It is discovered that the effect of diet on LTL differs greatly based on ethnicity [23], and Kiecoltglaser et al. [24] reports no significant effects of n-3 PUFA on telomere length. However, an intervention study indicates that replacing SFA with PUFA could have a very high impact on reducing the risk of cardiovascular disease [25], mainly because n-3 PUFA may lower the incidence of cardiovascular disease by slowing the rate of LTL abrasion [26]. There have also been researches showing that n-3 PUFA could slow the shortening rate of LTL, while n-6 PUFA could accelerate this rate [27]; these findings are contrary to the results of the present study. However, a randomized controlled trial indicates that treatment with n-3 PUFA do not lead to an increase in telomere length and that there is a trend toward telomere shortening during the intervention period in the population showing mild cognitive impairment [28]. Therefore, further investigation is urgently needed to understand the effects observed here, particularly to clarify whether RLTL is modified through a decrease in n-3 PUFA or (and/or) an increase in n-6 PUFA.

### HOMA-IR index and RLTL

HOMA-IR index is an indicator used to evaluate the level of insulin resistance, which is a pivotal factor affecting the occurrence of diabetes [29] and cardiovascular diseases [30], and a meta-analysis suggests that short LTL may be related to these diseases [31]. However, there is controversy among different scholars about the relation between HOMA-IR and LTL. LTL and HOMA-IR are negatively correlated with the female offspring of gestational diabetes mellitus and newly diagnosed type 2 diabetic patients [32, 33]. Aviv et al. [12] discovers that insulin resistance is inversely associated with LTL in premenopausal but not postmenopausal women. Barbieri et al. [10] found that age-adjusted LTL is not significantly associated with HOMA-IR. However, in present study, multiple models revealed that HOMA-IR was positively correlated with RLTL. Interestingly, a systematic review points out that insulin resistance and LTL occurrence exist in a vicious circle, such that insulin resistance, as a state of oxidative stress, could lead to faster LTL shortening, while LTL abrasion would further trigger or aggravate insulin resistance in turn [34]. It is, therefore, worth mentioning that the previous hypothesis that insulin resistance affects on LTL is limited to individuals diagnosed with insulin resistance or diabetes, whereas our study was aimed at evaluating this hypothesis in general populations and excluded diseases that impact insulin metabolism. Thus, the mechanism through which RLTL increases with increasing HOMA-IR index needs to be further explored.

### Interaction between HOMA-IR and elaidic acid on RLTL

It is well known that the combination effect of genetics background, biochemical and metabolic pathways, and behavioral lifestyles is the primary cause of mammalian aging. Telomeres are also influenced by these factors, acting as an indicator of biological aging [2]. This study showed that elaidic acid and HOMA-IR index are the possible LTL influencing factor. We, thus, further explored the interaction between elaidic acid and HOMA-IR index on RLTL. A similar study indicates that longer telomeres are related to lower white bread consumption and higher dietary total antioxidant capacity in Spanish children and adolescents [5]. Moreover, previous studies clearly show that consuming higher amounts of TFAs could cause or exacerbate insulin resistance in subjects with type 2 diabetes [35]. Therefore, it can be speculated that TFAs and insulin levels exhibit a synergistic effect. However, we discovered that the effects of HOMA-IR index and elaidic acid on RLTL may be antagonistic. Accordingly, these effects need to be researched further.

We would like to underline the novelty and limitations of the study. The first novelty of our study is its interaction design. Our research provides new insight into the effects of PPFAs and HOMA-IR index on RLTL among general human. Another novel feature of our study is that we used biomarkers instead of dietary fatty acids to predict the effects of PPFAs and HOMA-IR index on RLTL, which, to some extent, is more accurate and reliable. Furthermore, the relatively large sample size of our study allowed us to obtained results with statistical power in identifying significant associations of RLTL with PPFAs and HOMA-IR index. However, we also acknowledge some limitations of this study. First, our data were derived cross-sectionally from a general population and involved single RLTL measurement, which may not reflect telomere dynamics as well as repeated RLTL measurements, which would likely give more precise information. Second, due to the large sample size of this study, RLTL rather than absolute LTL was measured. Moreover, RLTL was determined for human peripheral blood leukocytes, only reflecting the mean telomere length of white cells. Finally, the change in telomere dynamics was mainly caused by oxidative stress and inflammatory response; however, owing to the lack of relative data, we only proved the relationship between PPFAs, HOMA-IR index, and RLTL. Although our current research has shortcomings, we believed that our results still provide helpful information regarding effects of PPFAs and HOMA-IR index on RLTL. Therefore, long term investigations of these effects are required to confirm the present findings and determine whether the results can be extended to other populations.

## Conclusion

In summary, we found PPFAs and HOMA-IR index to be associated with RLTL in the general population after adjusting for potential confounders. Particularly, shorter RLTL was found to be associated with a lower HOMA-IR index and a higher elaidic acid level.

## Supplementary information

**Additional file 1: Table S1.** Characteristics of subjects categorized by RLTL. **Table S2**. Correlation analysis of PPFAs and HOMA-IR with RLTL. **Table S3**. Multiple linear regression analysis of the association of PPFAs and HOMA-IR with RLTL among subjects. **Table S4**. Interaction between elaidic acid and HOMA-IR. Table S1 describes the characteristics of subjects by RLTL. The subjects were divided into tertiles according to RLTL (Tertiles cut-offs were 0.645, 1.884). Table S2 describes linear correlation of PPFAs and HOMA-IR with RLTL. All variables were log-transformed. Rude model represented that no confounding variables have been adjusted, and adjusted model only adjusted for age. Table S3 presents the multivariate linear regression results. All variables were log-transformed. Model 1 only adjusted for the age. Model 2 adjusted for the age, gender, race, smoking, drinking, tea and exercise and Model 3 adjusted for the BMI, WHR, SBP, DBP, TC, TG, and HDL-C on the basis of Model 2. Table S4 shows the effect of interaction between PPFAs and HOMA-IR on RLTL maintenance. As shown in Table 3, Model 1 represents the rude model and Model 2 only adjusted for the age. Model 3 adjusted for the age, gender, race, smoking, drinking, tea, and exercise, and Model 4 adjusted for the BMI, WHR, SBP, DBP, TC, TG, and HDL-C on the basis of Model 3.

## Data Availability

We declare that the data supporting the conclusions of this article are fully described within the article, and the database is available from the first author (zhaoyi751114@hotmail.com) upon reasonable request.

## Abbreviations

*95%CI*: 95% confidence intervals
BMI: Body mass index
DBP: Diastolic blood pressure
DHA: Docosahexaenoic acid
EPA: Eicosapentaenoic acid
HDL-C: High-density lipoprotein cholesterol
HOMA-IR: HOMA-insulin resistance
*IQR*: Interquartile range
LDL-C: Low-density lipoprotein cholesterol
LTL: Leukocyte telomere length
MUFA: Monounsaturated fatty acid
n-3: Omega-3
n-6: Omega-6
PPFAs: Plasma phospholipid fatty acids
PUFA: Polyunsaturated fatty acids
RLTL: Relative LTL
SBP: Systolic blood pressure
*SD*: Standard deviation
SFA: Saturated fatty acid
T_1_: Lowest tertile
T_2_: Middle tertile
T_3_: Upper tertiles
TC: Total cholesterol
TG: Triglycerides
WHR: Waist-to-hip ratio
*β*: Partial regression analysis
