# Supplementary material for: Functional interaction between plasma phospholipid fatty acids and insulin resistance in leucocyte telomere length maintenance
Source: Lipids Health Dis. 2020 Jan 17;19:11. doi: 10.1186/s12944-020-1194-1 (PMC6969400; doi:10.1186/s12944-020-1194-1)
Supplement: Supplementary file 1 — Additional file 1: Table S1. Characteristics of subjects categorized by RLTL. Table S2. Correlation analysis of PPFAs and HOMA-IR with RLTL. Table S3. Multiple linear regression analysis of the association of PPFAs and HOMA-IR with RLTL among subjects. Table S4. Interaction between elaidic acid and HOMA-IR. Table S1 describes the characteristics of subjects by RLTL. The subjects were divided into tertiles according to RLTL (Tertiles cut-offs were 0.645, 1.884). Table S2 describes linear correlation of PPFAs and HOMA-IR with RLTL. All variables were log-transformed. Rude model represented that no confounding variables have been adjusted, and adjusted model only adjusted for age. Table S3 presents the multivariate linear regression results. All variables were log-transformed. Model 1 only adjusted for the age. Model 2 adjusted for the age, gender, race, smoking, drinking, tea and exercise and Model 3 adjusted for the BMI, WHR, SBP, DBP, TC, TG, and HDL-C on the basis of Model 2. Table S4 shows the effect of interaction between PPFAs and HOMA-IR on RLTL maintenance. As shown in Table 3, Model 1 represents the rude model and Model 2 only adjusted for the age. Model 3 adjusted for the age, gender, race, smoking, drinking, tea, and exercise, and Model 4 adjusted for the BMI, WHR, SBP, DBP, TC, TG, and HDL-C on the basis of Model 3. [file 12944_2020_1194_MOESM1_ESM.doc]

Table S1 Characteristics of subjects categorized by RLTL

|  | All Subjects | RLTL | | | *P-value* |
| --- | --- | --- | --- | --- | --- |
|  |  | T_1_ (n = 415) | T_2_ (n = 415) | T_3_ (n = 416) |  |
| **General characteristics** |  |  |  |  |  |
| Gender (female) n (%) | 740 (59.4) | 231 (55.7) | 251 (60.5) | 258 (62.0) | 0.151 |
| Race(Han) n (%) | 697 (55.9) | 249 (60.0) | 221 (53.3) | 227 (54.6) | 0.116 |
| Age (years) | 50.04 ± 11.77 | 53.54 ± 10.82 | 49.37 ± 11.92 | 47.24 ± 11.70 | <0.001 |
| Smoking n (%) | 197 (15.8) | 65 (15.7) | 62 (14.9) | 70 (16.8) | 0.505 |
| Drinking n (%) | 130 (10.4) | 45 (10.8) | 51 (12.3) | 34 (8.2) | 0.572 |
| Tea n (%) | 654 (52.5) | 227 (54.7) | 236 (56.9) | 191 (45.9) | 0.004 |
| Exercise n (%) | 102 (8.2) | 41 (9.9) | 33 (8.0) | 28 (6.7) | 0.250 |
| BMI (kg/m^2^ | 23.57 ± 3.26 | 23.62 ± 3.41 | 23.53 ± 3.28 | 23.56 ± 3.09 | 0.914 |
| WHR | 0.88 ± 0.07 | 0.89 ± 0.07 | 0.88 ± 0.07 | 0.87 ± 0.07 | <0.001 |
| SBP (mmHg) | 127 ± 20 | 130 ± 20 | 127 ± 20 | 125 ± 21 | 0.004 |
| DBP (mmHg) | 80 ± 12 | 80 ± 12 | 79 ± 11 | 80 ± 12 | 0.310 |
| **Metabolic indicators** |  |  |  |  |  |
| Fasting Plasma Insulin (mU/L)* | 5.39 (3.49, 6.69) | 4.96 (2.73, 6.47) | 5.26 (3.19, 6.68) | 5.64 (4.93, 6.90) | <0.001 |
| Fasting Plasma Glucose (mmol/L) | 5.73 ± 1.01 | 5.69 ± 0.74 | 5.90 ± 1.42 | 5.60 ± 0.68 | <0.001 |
| HOMA-IR* | 1.32 (0.90, 1.72) | 1.23 (0.70, 1.65) | 1.32 (0.83, 1.72) | 1.38 (1.17, 1.78) | <0.001 |
| TC (mmol/L) | 3.97 ± 0.84 | 4.02 ± 0.80 | 3.97 ± 0.85 | 3.93 ± 0.87 | 0.283 |
| TG (mmol/L) | 1.38 ± 0.88 | 1.37 ± 0.74 | 1.39 ± 0.94 | 1.40 ± 0.94 | 0.937 |
| HDL-Cholesterol (mmol/L) | 1.35 ± 0.32 | 1.38 ± 0.30 | 1.37 ± 0.32 | 1.30 ± 0.34 | <0.001 |
| LDL-Cholesterol (mmol/L) | 2.00 ± 0.66 | 2.02 ± 0.65 | 1.98 ± 0.65 | 2.00 ± 0.68 | 0.709 |
| **PPFAs*** |  |  |  |  |  |
| Palmitic acid (ng/mL) | 47.70 (31.92, 73.49) | 52.49 (34.72, 80.79) | 49.18 (32.41, 74.04) | 43.98 (29.46, 69.25) | 0.002 |
| Stearic acid (ng/mL) | 42.87 (29.93, 60.43) | 45.66 (32.00, 65.10) | 43.47 (30.10, 58.30) | 39.64 (27.65, 55.96) | 0.005 |
| Elaidic acid (ng/mL) | 85.11 (58.62, 117.85) | 90.75 (67.67, 130.78) | 86.55 (61.99, 114.89) | 77.53 (46.58, 108.09) | <0.001 |
| Linoleic acid (ng/mL) | 56.78 (40.12, 79.67) | 56.59 (41.92, 80.58) | 56.13 (39.70, 72.77) | 58.33 (39.09, 85.59) | 0.261 |
| α-Linolenic acid (ng/mL) | 4.98 (2.83, 9.33) | 5.70 (3.45, 9.96) | 5.16 (3.16, 9.48) | 3.98 (2.06, 8.18) | <0.001 |
| Arachidonic acid (ng/mL) | 22.10 (14.71, 31.48) | 24.44 (16.06, 32.58) | 21.35 (15.22, 29.75) | 21.33 (13.34, 31.42) | 0.009 |
| EPA (ng/mL) | 3.41 (2.51, 4.96) | 2.70 (3.78, 5.20) | 3.23 (2.47, 4.78) | 3.18 (2.23,5.26) | 0.045 |
| DHA (ng/mL) | 5.48 (3.95, 7.78) | 5.56 (4.03, 8.45) | 5.59 (4.00, 7.52) | 5.28 (3.78, 7.24) | 0.123 |
| SFA (ng/mL) | 91.84 (63.40, 131.83) | 98.70 (68.14, 140.84) | 92.56 (65.51, 132.00) | 85.06 (58.31, 125.21) | 0.001 |
| MUFA (ng/mL) | 89.38 (66.18, 125.20) | 94.24 (71.08, 133.13) | 89.03 (65.48, 118.96) | 85.52 (60.24, 124.70) | 0.010 |
| PUFA (ng/mL) | 91.56 (64.88, 125.87) | 96.08 (70.08, 129.63) | 88.82 (63.67, 119.08) | 91.42 (60.20, 128.14) | 0.090 |
| n-6 PUFA (ng/mL) | 79.48 (57.41, 108.31) | 80.98 (61.24, 112.60) | 77.03 (57.20, 101.92) | 80.32 (55.19, 112.92) | 0.125 |
| n-3 PUFA (ng/mL) | 10.67 (4.93, 18.19) | 11.90 (6.58, 20.04) | 10.98 (4.74, 18.13) | 9.64 (3.32, 16.55) | <0.001 |
| n-6/n-3 | 7.32 (4.74, 11.92) | 6.63 (4.56, 10.57) | 7.05 (4.77, 11.12) | 8.27 (4.99, 14.08) | <0.001 |

Abbreviations: RLTL, relative leukocyte telomere length; BMI, body mass index; WHR, waist-to-hip ratio; SBP, systolic blood pressure; DBP, diastolic blood pressure; TC, total cholesterol; TG, triglycerides; HDL-C, high-density lipoprotein cholesterol; LDL-C, low-density lipoprotein cholesterol; PPFAs, plasma phospholipid fatty acids, EPA, eicosapentaenoic acid; DHA, docosahexaenoic acid; SFA, saturated fatty acid; MUFA, monounsaturated fatty acid; PUFA, polyunsaturated fatty acids; T_1_, lowest tertile; T_2_, middle tertile; T_3_, upper tertiles; n, number

* Values shown are Median (*P25*, *P75*).

Table S2 Correlation analysis of PPFAs and HOMA-IR with RLTL

|  | RLTL | | | | | |  |
| --- | --- | --- | --- | --- | --- | --- | --- |
|  | Rude Model | |  | | Adjusted Model***** | |  |
|  | *r* | *P-value* | |  | *r* | *P-value* | |
| Palmitic acid (ng/mL) | -0.089 | 0.002 | |  | -0.088 | 0.002 | |
| Stearic acid (ng/mL) | -0.080 | 0.005 | |  | -0.081 | 0.004 | |
| Elaidic acid (ng/mL) | -0.170 | <0.001 | |  | -0.158 | <0.001 | |
| Linoleic acid (ng/mL) | -0.027 | 0.334 | |  | -0.029 | 0.311 | |
| α-Linolenic acid (ng/mL) | -0.133 | <0.001 | |  | -0.099 | 0.001 | |
| Arachidonic acid (ng/mL) | -0.067 | 0.019 | |  | -0.071 | 0.013 | |
| EPA(ng/mL) | -0.016 | 0.706 | |  | -0.026 | 0.535 | |
| DHA(ng/mL) | -0.049 | 0.169 | |  | -0.051 | 0.155 | |
| SFA(ng/mL) | -0.086 | 0.002 | |  | -0.085 | 0.003 | |
| MUFA(ng/mL) | -0.095 | 0.001 | |  | -0.087 | 0.002 | |
| PUFA(ng/mL) | -0.055 | 0.053 | |  | -0.052 | 0.066 | |
| n-6 PUFA (ng/mL) | -0.040 | 0.163 | |  | -0.041 | 0.147 | |
| n-3 PUFA (ng/mL) | -0.107 | <0.001 | |  | -0.082 | 0.005 | |
| n-6/n-3 | 0.112 | <0.001 | |  | 0.078 | 0.008 | |
| TC (mmol/L) | -0.075 | 0.008 | |  | 0.005 | 0.855 | |
| TG (mmol/L) | -0.060 | 0.033 | |  | -0.023 | 0.416 | |
| HDL-Cholesterol (mmol/L) | -0.102 | <0.001 | |  | -0.057 | 0.045 | |
| LDL-Cholesterol (mmol/L) | -0.036 | 0.202 | |  | 0.028 | 0.320 | |
| Fasting Plasma Insulin (mU/L) | -0.040 | 0.156 | |  | -0.012 | 0.679 | |
| Fasting Plasma Glucose (mmol/L) | 0.153 | <0.001 | |  | 0.132 | <0.001 | |
| HOMA-IR | 0.135 | <0.001 | |  | 0.122 | <0.001 | |

Abbreviations: *r*, correlation coefficient; ***** adjusted for age; All variables were log-transformed.

Table S3 Multiple linear regression analysis of the association of PPFAs and HOMA-IR with RLTL among subjects

| Variables | Model 1 | |  | Model 2 | |  | Model 3 | |
| --- | --- | --- | --- | --- | --- | --- | --- | --- |
|  | *β (95%CI)* | *P-value* |  | *β (95%CI)* | *P-value* |  | *β (95%CI)* | *P-value* |
| Palmitic acid | -0.031 (-0.269, 0.207) | 0.800 |  | 0.001 (-0.240, 0.241) | 0.996 |  | -0.043 (-0.293, 0.208) | 0.738 |
| Stearic acid | 0.094 (-0.142, 0.331) | 0.434 |  | 0.083 (-0.154, 0.320) | 0.493 |  | 0.095 (-0.148, 0.338) | 0.444 |
| Elaidic acid | -0.425 (-0.603, -0.248) | <0.001 |  | -0.398 (-0.578, -0.219) | <0.001 |  | -0.401 (-0.584, -0.218) | <0.001 |
| α-Linolenic acid | 0.002 (-0.150, 0.155) | 0.975 |  | -0.003 (-0.158, 0.152) | 0.967 |  | -0.001 (-0.159, 0.157) | 0.991 |
| Arachidonic acid | 0.043 (-0.147, 0.234) | 0.657 |  | 0.022 (-0.169, 0.214) | 0.819 |  | 0.053 (-0.142, 0.248) | 0.595 |
| HOMA-IR | 0.288 (0.153, 0.422) | <0.001 |  | 0.289 (0.153, 0.424) | <0.001 |  | 0.264 (0.122, 0.406) | <0.001 |
| SFA | -0.123 (-0.319, 0.072) | 0.216 |  | -0.121 (-0.317, 0.075) | 0.225 |  | -0.142 (-0.339, 0.054) | 0.156 |
| MUFA | -0.302 (-0.545, -0.059) | 0.015 |  | -0.230 (-0.478, 0.018) | 0.069 |  | -0.224 (-0.478, 0.029) | 0.083 |
| n-6 PUFA | 0.371 (0.123, 0.619) | 0.003 |  | 0.379 (0.130, 0.627) | 0.003 |  | 0.395 (0.142, 0.649) | 0.002 |
| n-3 PUFA | -0.126 (-0.257, 0.004) | 0.058 |  | -0.160 (-0.293, -0.027) | 0.018 |  | -0.155 (-0.290, -0.020) | 0.025 |
| HOMA-IR | 0.294 (0.161, 0.426) | <0.001 |  | 0.295 (0.161, 0.429) | <0.001 |  | 0.277 (0.136, 0.419) | <0.001 |

Abbreviations: *β*, partial regression analysis; *CI*: confidence interval; All variables were log-transformed

Model 1: adjusted for age; Model 2: Model 1 plus gender, race, smoking, drinking, tea, exercise; Model 3: adjusted for all the confounders in Model 2 as well as BMI, WHR, SBP, DBP, TC, TG, HDL-C.

Table S4 Interaction between elaidic acid and HOMA-IR

| Variables | Model 1 | |  | Model 2 | |  | Model 3 | |  | Model 4 | |
| --- | --- | --- | --- | --- | --- | --- | --- | --- | --- | --- | --- |
|  | *F* | *P-value* |  | *F* | *P-value* |  | *F* | *P-value* |  | *F* | *P-value* |
| elaidic acid | 14.802 | <0.001 |  | 12.325 | <0.001 |  | 9.170 | <0.001 |  | 9.186 | <0.001 |
| HOMA-IR | 26.874 | <0.001 |  | 18.372 | <0.001 |  | 18.648 | <0.001 |  | 15.921 | <0.001 |
| elaidic acid * HOMA-IR | 2.927 | 0.020 |  | 2.467 | 0.043 |  | 2.456 | 0.044 |  | 2.491 | 0.042 |

Model 1: rude model; Model 2: adjusted for age; Model 3: Model 2 plus gender, race, smoking, drinking, tea, exercise; Model 4: adjusted for all the confounders in Model 3 as well as BMI, WHR, SBP,

DBP, TC, TG, HDL
